# *Lobesia botrana* Larvae Develop Faster in the Presence of Parasitoids

**DOI:** 10.1371/journal.pone.0072568

**Published:** 2013-08-28

**Authors:** Fanny Vogelweith, Yannick Moret, Denis Thiery, Jérôme Moreau

**Affiliations:** 1 Université de Bourgogne, Equipe Ecologie Evolutive, UMR 6282 Biogéosciences, Dijon, France; 2 INRA UMR 1065 Santé et Agroecologie du Vignoble, Institut des Science de la Vigne et du Vin, Villenave d’Ornon Cedex, France; 3 Université de bordeaux, INRA UMR 1065, Save, Bordeaux Sciences Agro, Villenave d’Ornon Cedex, France; Ghent University, Belgium

## Abstract

To combat parasitism hosts often rely on their immune system, which is the last line of defense. However, the immune system may not always be effective, and other non-immunological defenses might be favored to reduce the cost of parasite infection. Here we report that larvae of the moth *Lobesia botrana* can rapidly accelerate their development and reach maturity earlier in response to cues perceived at a distance from parasitoids. Such a phenotypically plastic life history shift, induced by the perception of deadly enemies in the environment, is likely to be an adaptive defensive strategy to prevent parasitoid attack, and has important implications in host–parasite dynamics.

## Introduction

Parasites are omnipresent and can dramatically impact host growth, survival and reproduction, which together determine host life history [1], [2]. When threatened by parasite infection, the host can reduce its loss of fitness by using its immune system to control the parasite. However, the immune system of the host is not always effective, and as an alternative it can reduce adverse parasite effects by facultative adjustment of its life history parameters [3], [4], [5]. For example, freshwater snails and crustaceans infected by castrating parasites reach reproductive maturity earlier [3], [5], ensuring production of some offspring before the effects of castration establish.

Such parasite-induced life history transitions often result in the reallocation of resources from growth to reproduction [6]. Consequently, hosts that mature earlier are smaller, which is often correlated with low fecundity and reduced longevity [1], [2]. The animal kingdom includes numerous vertebrate [7] and invertebrate [8] correlations examples of pre-reproductive life span with adult size and fecundity. Thus, as accelerated development imposes significant costs, the host should trigger life history transitions only when highly reliable cues indicate an imminent and severe parasitic infection.

Parasite cues inducing life history transitions are often associated with the infection event [3], [5] or stimulation of the host immune system [9], [10]. However, hosts can benefit from shifting their life histories prior to infection, as this reduces the overall cost of infection. This requires that host can highly reliably sense cues predicting infection prior to its occurrence. For instance, life history changes were induced in freshwater snails by exposing them to water that contained a parasite, in the absence of infection [6].

For holometabolous herbivorous insects total enemy-induced mortality is higher during late developmental stages, and larval parasitoids kill more herbivores than do either predators or pathogens [11]. Therefore, parasitoids represent a major selective force shaping defensive strategies during larval stages of these insects. As the evolution of immune resistance to parasitoid attack has substantial constitutive costs [12], plastic shortening of the pre-reproductive life stages (i.e. reaching metamorphosis earlier) in response to cues indicating imminent larval parasitoid attack is likely to be favored among insect hosts. However, because early maturation is costly, representing a decrease in reproductive output, insect larvae are expected to have evolved highly accurate recognition of cues specific to parasitoids.

In this study we assessed this hypothesis using the moth *Lobesia botrana* (a major grapevine pest) as a holometabolous herbivorous insect model system. Natural populations of *L. botrana* are the targets of numerous species of parasitoids [13]. These populations face variable temporal and spatial changes in parasitoid pressure, which determine temporally and spatially variable risks of attacks [14]. Here, we mimicked imminent parasitoid attacks by exposing larvae of *L. botrana* to the presence of parasitoid and non-parasitoid insects without physical contact, and monitored the time for these larvae to reach metamorphosis. If *L. botrana* larvae could sense cues indicating the presence of parasitoid, we expect they will shorten their larval development and reach metamorphosis earlier. Because the shift toward earlier maturation is costly, we expect that *L. botrana* larvae will manage to differentiate cues from parasitoid and non-parasitoid insects and accelerate their larval development only in the presence of parasitoids.

## Materials and Methods

### Ethic Statement

This study conformed to French legal requirements and to accepted international ethical standards including those relating to conservation and welfare, and to the journal’s policy on these matters. All experiments complied with French laws guiding the care and use of animals.

*Lobesia botrana* (Lepidoptera: Tortricidae) is one of the major grape pests in Europe. Following hatching the larvae grow through a succession of five instar stages before metamorphosis and emergence. The larvae used in this study were descended from individuals that were collected in a Sauternes vineyard in western France and used to initiate a laboratory culture maintained at the French National Institute for Agricultural Research (INRA; Bordeaux, France). The larvae were maintained in boxes (18×11.5×7 cm) under standard laboratory conditions (22±1°C; 70±10% relative humidity; photoperiod: L16:D8) with *ad libitum* supply of a semi-artificial diet (see [15]) at a density of 100 individuals per 300 ml of diet.

Two species of parasitoid, *Campoplex capitator* (Hymenoptera: Ichneumonidae) and *Phytomyptera nigrina* (Diptera: Tachinidae), were used to mimic imminent parasitoid attack. These parasitoids parasitize the L3 and L4 larvae of *L. botrana* [13]. They were obtained from parasitized *L. botrana* chrysalises collected in a vineyard in Perpignan (southern France).

Because this life history response is likely costly, we may expect that high degree of specificity could have been selected in *L. botrana*. For instance, one could expect that larvae would not accelerate their development when exposed to parasitoid insects that are phylogenetically close to their actual parasitoids but that do not parasitize *L. botrana*. Unfortunately, such non parasiting parasitoid has not yet being reported for *L. botrana*. Therefore, the vomiting green bottle fly, *Lucilia caesar* (Diptera: Calliphoridae), was used as a non-parasitoid insect for a control. The flies were obtained from a commercial source (Decathlon, Dijon, France).

Fourth instar larvae of *L. botrana* were exposed in groups of 5 larvae to one of the parasitoids (*C. capitator* or *P. nigrina*), the non-parasitoid fly (*L. caesar*), or to no insect. For these experiments the *L. botrana* larvae were placed in plastic boxes (98×98×49 mm) containing 50 ml of semi-artificial diet [15]. A plastic cup (30 mm diameter×30 mm height) containing one adult of *C. capitator* (n = 15), *P. nigrina* (n = 15), *L. caesar* (n = 15), or no insect (n = 19) was placed in the middle of each box. The central cup was transparent and had been pierced to produce many small holes, facilitating visual, olfactory and vibratory stimulation of the *L. botrana* larvae by the test insects. The test insects were fed with a drop of honey for the duration of the experiment. The experimental boxes were maintained in standard laboratory conditions (22±1°C; 70±10% relative humidity; photoperiod: L16:D8). After six days the larvae in each box were scored as ‘dead’, ‘live’ or ‘chrysalis’.

All statistical tests were performed using the R 2.15.0 (R Development Core Team 2012) software. With our experimental procedure, we do not consider each individual as an independent replicate. Therefore, interpretations of the differences between mean proportions of dead, live or chrysalis among treatments (absence/presence of *C. capitator*, *P. nigrina* or *L. caesar*) were made using the 95% confidence interval (C.I.) and bootstrapped data. Significant differences among treatments were assessed by comparison of confidence intervals [16].

## Results

**Figure 1:**
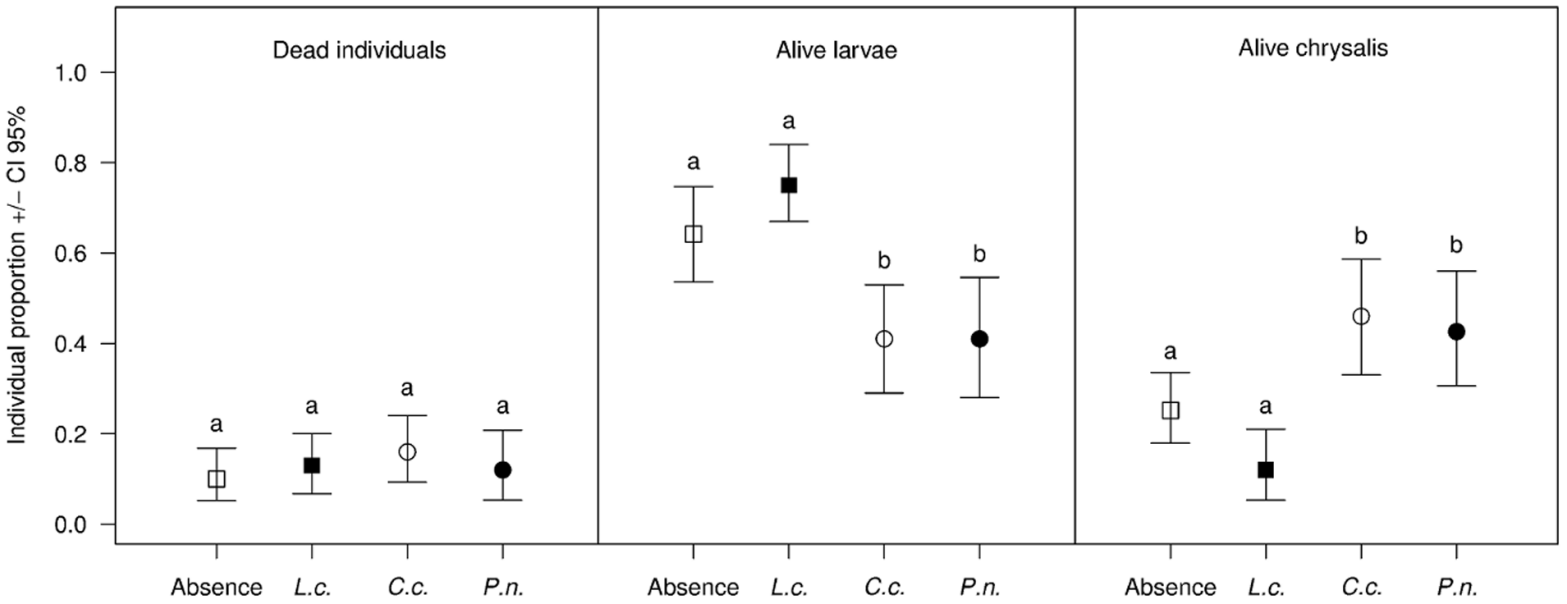
Response of *Lobesia botrana* larvae to parasitoid presence. Mean proportions of dead individuals, alive larvae and chrysalises of *Lobesia botrana* (C.I. 95%) following six days in the presence of the non-parasitoid *Lucilia caesar* (*L.c.*; black squares), the parasitoid *Campoplex capitator* (*C.c.*; white circle) or *Phytomyptera nigrina* (*P.n.*; black circle), or in the absence (white squares) of any insect. Different letters represent significant differences within each category of *L. botrana*.

Thirteen per cent of the *L. botrana* larvae died during the experiment, and the mortality was independent of the threat treatment (Fig. 1).

At the conclusion of the experiment the proportion of live larvae was higher in treatments lacking insects (64%) or containing *L. caesar* (75%) than in treatments involving the parasitoid *C. capitator* or *P. nigrina* (41% each). In contrast, the proportion of chrysalises was higher in the presence of *C. capitator* (46%) and *P. nigrina* (42%) than in the presence of *L. caesar* (12%) or the absence of any insect (25%) (Fig. 1).

## Discussion

Parasitoid threat elicited expression of phenotypic plasticity during *L. botrana* larval development, resulting in the larvae reaching metamorphosis earlier. Similar phenomena induced by natural parasite infection [3], [5], [6] and artificial immune stimulation [9], [10] have been reported previously. Host physiology and behavior can be dramatically altered following infection, through the action of the parasite or/and the action of the immune system [17], a secondary effect of which could be a shortening of larval development. Our experimental setting prevented any contact of the moth larvae with the parasitoids, and therefore excluded any direct effect of parasitoid infection or associated response of the host immune system. Therefore, the observed effects probably reflect a plastic life history adjustment triggered by detection of an imminent parasitoid attack.

By shortening larval development moths may escape potential attack by parasitoids. Hard external chrysalis teguments are likely to be resistant to perforation by the parasitoid ovipositor, and the adults are able to escape by flight. In addition, most parasitoids (including those that parasitize *L. botrana*) infect their hosts at the larval, and not the chrysalis, stage [15]. Hence, a life history change shortening larval development, induced by the presence of parasitoids, is probably an adaptive defensive strategy against parasitoid attack.

The development of larvae of *L. botrana* was not altered by the presence of a non-parasitoid insect. This suggests that plastic shortening of larval development is a specific response to parasitoids, which may be a strategy to prevent the unnecessary triggering of accelerated larval development, and avoiding the associated life history costs. Such specificity supports the hypothesis that the parasitoid-induced life history change in *L. botrana* is a defensive strategy that has evolved in response to selective pressure imposed by parasitoids.

Our results further highlight that *L. botrana* larvae are able to assess the risk of parasitoid attack in the absence of physical contact. We did not aim to identify the processes used by the larvae to sense threats in their environment, as our experimental design focused on olfactory, visual and vibratory stimuli. While these senses could be involved in discriminating conspecifics and predators in other phytophagous insects [18], [19], further study will be needed to estimate the relative contributions of incoming sensory signals that are used as cues to assess parasitoid threat. Moreover, molecular and physiological mechanisms by which incoming sensory signals lead to life history changes should be of great importance in understanding how the environment shapes phenotype.

The moth *L. botrana* specifically reacted to cues indicating the presence of parasitoids with a phenotypically plastic life history shift involving accelerated development, such that maturity was reached earlier. This suggests that shifts in host life history can be triggered in the absence of physical contact with parasitoids. The observed phenomenon could not be explained by a direct effect of parasitoid infection or a response of the host immune system. We believe that such plasticity in life history traits in response to cues perceived at a distance is adaptive, and has significant implications for our understanding of population dynamics between phytophagous insects, their host plants and their natural enemies.
